# Significant SNPs have limited prediction ability for thyroid cancer

**DOI:** 10.1002/cam4.211

**Published:** 2014-03-03

**Authors:** Shicheng Guo, Yu-Long Wang, Yi Li, Li Jin, Momiao Xiong, Qing-Hai Ji, Jiucun Wang

**Affiliations:** 1 State Key Laboratory of Genetic Engineering and Ministry of Education Key Laboratory of Contemporary Anthropology, School of Life Sciences, Fudan UniversityShanghai, 200433, China; 2 Human Genetics Center, University of Texas School of Public HealthHouston, Texas, 77030; 3 Department of Head and Neck Surgery, Fudan University Shanghai Cancer CenterShanghai, 200032, China; 4 Department of Oncology, Shanghai Medical College, Fudan UniversityShanghai, 200032, China; 5 Fudan-Taizhou Institute of Health SciencesTaizhou, Jiangsu, 225300, China

**Keywords:** Genetic, risk prediction, SNPs, thyroid cancer

## Abstract

Recently, five thyroid cancer significantly associated genetic variants (rs965513, rs944289, rs116909374, rs966423, and rs2439302) have been discovered and validated in two independent GWAS and numerous case–control studies, which were conducted in different populations. We genotyped the above five single nucleotide polymorphisms (SNPs) in Han Chinese populations and performed thyroid cancer-risk predictions with nine machine learning methods. We found that four SNPs were significantly associated with thyroid cancer in Han Chinese population, while no polymorphism was observed for rs116909374. Small familial relative risks (1.02–1.05) and limited power to predict thyroid cancer (AUCs: 0.54–0.60) indicate limited clinical potential. Four significant SNPs have limited prediction ability for thyroid cancer.

## Introduction

Thyroid cancer is the fifth most common type of female cancer and its incidence is increasing. It has been considered as one of highest familial risk carcinomas among all kinds of cancers 1,2. Most common diseases are caused by multiple genetic rather than few loci. In the last 2 years, two independent genome-wide association studies (GWAS) have been conducted to identify single nucleotide polymorphisms (SNPs) associated with thyroid cancer risk. Five SNPs (rs965513, rs944289, rs116909374, rs966423, and rs2439302) which were highly significantly associated with papillary thyroid carcinoma (PTC) were discovered by genome-wide association study. In addition, these five SNP were validated by continued case–control studies in more than three different populations (Han Chinese, Ohio, Poland, etc. Table 1).

**Table 1:** Odds ratio for five SNPs from GWAS and case–control association study in previous study

			OR (*P*-value)^1^,^2^					
				
Study	Population	Method	rs965513	rs944289	rs116909374	rs966423	rs2439302	Reference
1^1^	Iceland	GWAS	1.73 (7.5e-13)	1.48 (8.6e-7)	–	–	–	12
	Iceland all	Combined	1.77 (6.8e-20)	1.44 (2.5e-8)				
	USA	Case–control	1.81 (1.2e-7)	1.32 (1.2e-2)				
	Spain	Case–control	1.54 (6.5e-3)	1.14 (4.3e-1)				
	USA and Spain	Case–control	1.72 (3.7e-9)	1.26 (1.1e-2)				
	All combined	Combined	1.75 (1.7e-27)	1.37 (2.0e-9)				
2^1^	Chernobyl	GWAS	1.76 (4.9e-9)	1.13 (0.17)	–	–	–	13
	Combined	1.65 (4.8e-12)	–					
3^2^	Japan	Case–control	1.69 (1.27e-4)	1.21 (0.0121)	–	–	–	14
4^2^	UK	Case–control	1.98 (6.35e-34)	1.33 (6.95e-7)	–	–	–	15
5^1^	Iceland	Case–control	1.70 (3.0e-18)	1.36 (4.2e-5)	2.03 (5.4e-7)	1.26(3.8e-4)	1.41 (1.3e-6)	16
	Netherland	Case–control	–	1.39 (0.013)	1.95 (0.024)	1.80(4.2e-6)	1.24 (0.088)	
	USA	Case–control	–	1.51 (0.0067)	1.98 (0.018)	1.36 (3.5e-3)	1.33 (6.1e-3)	
	Spain	Case–control	–	1.17 (0.31)	3.37 (2.6e-3)	1.20 (0.24)	1.34 (0.073)	
	All combined	Case–control	–	1.36 (4.9e-8)	2.09 (4.6e-11)	1.34 (1.3e-9)	1.36 (2.0e-9)	
6^1^	USA	Case–control	2.10 (<2e-16)	1.28 (1.99e-3)	1.97 (1.11e-3)	1.35 (1.75e-4)	1.51 (4.24e-7)	11
	Poland	Case–control	1.78 (<2e-16)	1.21 (3.55e-3)	1.73 (6.27e-3)	1.15 (3.13e-2)	1.27 (2.20e-4)	
7^1^,^2^	China	Case–control	1.53^1^ (7.1e-4)	1.51^1^ (2.8e-9)	–	1.32^1^ (0.006)	1.40^1^ (2.1e-4)	3
			1.53^2^ (1.4e-4)	1.53^2^ (2.0e-10)		1.31^2^ (0.001)	1.41^2^ (2.7e-5)	

GWAS, genome-wide association studies; OR, odds ratio.

1 ORs were calculated based on the multiplicative model. For the combined study populations, the OR value were estimated using the Mantel–Haenszel model.

2 ORs were calculated for the risk allele with using multiple logistic regression analyses.

To examine the prediction ability based on variants with highly significant associations, we use all five SNPs to predict thyroid cancer by nine classification methods (K-nearest neighbors, logistic regression, naïve Bayes, random forest, support vector machine, Bayesian additive regression trees (BART), recursive partitioning, fuzzy rule-based system, boosting). Contradictory to our intuitiveness, we found that although all these five SNPs were significantly associated with thyroid cancer, the precision of their prediction for thyroid cancer was very low.

## Methods

The five SNPs were genotyped in 845 PTC and 1005 controls in Han Chinese population using the SNaPshot multiplex single-nucleotide extension system. PTC patients who were treated in the Department of Head and Neck Surgery, Fudan University Shanghai Cancer Center, Shanghai, China from January to December 2010 were enrolled in this study. All patients were ethnically Chinese Han and came from Eastern China. A total of 1005 cancer-free unrelated individuals were recruited from the Taizhou Longitudinal Study (TZL). The SNPs were genotyped with the SNaPshot multiplex single-nucleotide extension system. Details of SNPs (Table S1) and primers were listed in our previous article 3.

The relative risk to daughters of an affected thyroid cancer individual attributable to a given SNP is calculated by the formula: 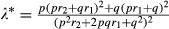, where *p* is the frequency of the risk allele, *q* = 1 − *p*, *r*_1_ and *r*_2_ are the relative risks (estimated by odds ratio [ORs]) for heterozygotes relative to common homozygotes and rare homozygotes relative to common homozygotes in the population, respectively 4,5. Assuming a multiplicative interaction, the proportion of the familial risk attributable to the SNP is calculated by log(λ*)/log(*λ*_o_), where *λ*_o_ is the overall familial relative risk (FRR), estimated to be 8.48 for thyroid cancer 1. Gender- and age-matched cases and controls were constructed by 1000 times resampling technology.

Nine machine learning methods were used to make prediction for PTC from health individuals, including K-nearest neighbors 6, logistic regression, naïve Bayes 7, random forest 8, support vector machine 7, BART 9, boosting, recursive partitioning, and fuzzy rule-based system 10. The parameters in the models were optimally selected. Classification accuracy, sensitivity, specificity, and AUC were used to evaluate the performance of the methods. They were calculated by 10-fold cross-validation.

## Results

### Marginal FRR of the significant SNPs

As the previous studies showed that the five SNPs with large OR were significantly associated with thyroid cancer in various populations (Table 1). Our previous data also showed that SNPs were significantly associated with thyroid cancer in Chinese population (the seventh study of Table 1). In present study, we estimated the FRR for five significantly associated SNPs in Chinese population. We found that the FRRs were low, ranging from 1.02 to 1.05. These five SNPs counted only 5.98% of the overall familial risk (Table 2) which was very closed to that of polish population (about 6%) 11. Our finding suggested that majority of the heritability was undiscovered.

**Table 2:** Estimation of familial relative risk of thyroid cancer for the five SNPs in population of Han Chinese

SNPs	Familial relative risk	Proportion (100%)	*P*-value
rs965513	1.0189 (1.0186–1.0192)	0.843 (0.806–0.880)	<2.2e-16
s944289	1.0419 (1.0415–1.0422)	1.969 (1.922–2.016)	<2.2e-16
rs116909374	N.A.^1^	N.A.^1^	N.A.^1^
rs966423	1.0493 (1.0485–1.0500)	2.191 (2.093–2.289)	<2.2e-16
rs2439302	1.0207 (1.0205–1.0210)	0.977 (0.939–1.015)	<2.2e-16

1 rs116909374 SNP was not detected in the Chinese population.

### Genetic risk prediction for thyroid cancer based on five SNPs

The five significant SNPs were used to predict risk of thyroid cancer by nine classification methods. The results were summarized in Table 3. The prediction accuracies ranged from 0.52 to 0.57 in the nine prediction methods, while receiver operating characteristics (ROCs) ranged from 0.54 to 0.60. The sensitivity of the prediction (0.28–0.48) was much less than specificity (0.56–0.76), which suggested the clinical application value might be limited (Table 3). In addition, the AUC of classification based on five SNPs and gender, and based on five SNPs, gender, and age ranged from 0.49 to 0.58, and from 0.50 to 0.59, respectively. This indicated that including gender and age information will not improve prediction (Tables S2 and S3, Fig. S1).

**Table 3:** Model performance with methods based on five significant SNPs

	AUC	Sensitivity	Specificity	Accuracy	Range of 95% CI of AUC
K-nearest neighbors	0.5589	0.3861	0.6591	0.533	[0.4293, 0.7101]
Logistic regression	0.6044	0.4982	0.5648	0.5346	[0.4433, 0.7368]
Naïve Bayes	0.5996	0.3921	0.7206	0.5686	[0.4571, 0.7469]
Random forest	0.5743	0.3169	0.7558	0.5535	[0.4405, 0.7233]
Support vector machine	0.5494	0.2762	0.7775	0.547	[0.4187, 0.7086]
Bayesian additive regression trees	0.5906	0.4779	0.5571	0.5211	[0.4385, 0.7211]
Boosting	0.6024	0.4723	0.5544	0.5157	[0.4584, 0.7287]
Recursive partitioning	0.5871	0.4085	0.7218	0.5778	[0.3926, 0.7048]
Fuzzy rule-based system	0.5396	0.4931	0.5006	0.4968	[0.4115, 0.6710]

AUC, sensitivity, specificity, and accuracy were its mean value in 10-fold validations. Range of 95% CI of AUC represents the range of the 95% CI of AUC in 10-fold Cross-validation. SVM represents support vector machines and Kernel Methods.

## Conclusion

In the present study, we estimated the FRR and evaluated thyroid cancer prediction accuracy of the five SNPs that showed significant association with thyroid cancer in several association studies. The results showed that although the OR of each SNPs was large, the FRR of each SNPs was very marginal. By 10-fold cross-validation, we found that the prediction accuracy of five SNPs was low across all nine classification methods. Particularly, the sensitivity of five SNPs was very low. It suggested that the clinical application of five SNPs might be limited. Our results strongly demonstrate that complex diseases are caused by a large number of SNPs, environments, and their interactions. GWAS addressing common variants have come to its limit and missing heritability for most complex disorders is very high. Only about 5–10% heritability was found based on common disease common variant (CDCV) model. To improve prediction of genetic variation for complex diseases, we need to incorporate more common and rare SNPs, copy number variations (CNVs), and nongenetic susceptibility factors, such as iodine intake, exposure to radiation in the classification analysis. Novel statistical methods for variable screening should be developed to optimally select SNPs and CNVs across the genome for disease risk prediction.

## Supporting Information

**Figure S1.** ROC comparison among all the machine learning prediction methods. Nine machine learning method were used to make prediction for PTC from health individuals, including K-nearest neighbors (KNN), logistic regression (LR), naïve Bayes, random forest, support vector machine, Bayesian additive regression trees (BART), boosting, recursive partitioning, fuzzy rule-based system. The parameters in the models were optimally selected. Classification accuracy, sensitivity, specificity and AUC were used to evaluate the performance of the methods. They were calculated by 10-fold cross-validation.

**Table S1.** Genomic information for five Acknowledged SNPs from GWAS. **Table S2.** Model performance with methods based on five SNPs and gender. **Table S3.** Model performance with methods based on five SNPs, gender, and age.
