# Supplementary material for: Significant SNPs have limited prediction ability for thyroid cancer
Source: Cancer Med. 2014 Mar 3;3(3):731–5. doi: 10.1002/cam4.211 (PMC4101765; doi:10.1002/cam4.211)
Supplement: Supplementary file 1 — Figure S1. ROC comparison among all the machine learning prediction methods. Nine machine learning method were used to make prediction for PTC from health individuals, including K-nearest neighbors (KNN), logistic regression (LR), naïve Bayes, random forest, support vector machine, Bayesian additive regression trees (BART), boosting, recursive partitioning, fuzzy rule-based system. The parameters in the models were optimally selected. Classification accuracy, sensitivity, specificity and AUC were used to evaluate the performance of the methods. They were calculated by 10-fold cross-validation. [file cam40003-0731-SD1.pdf]

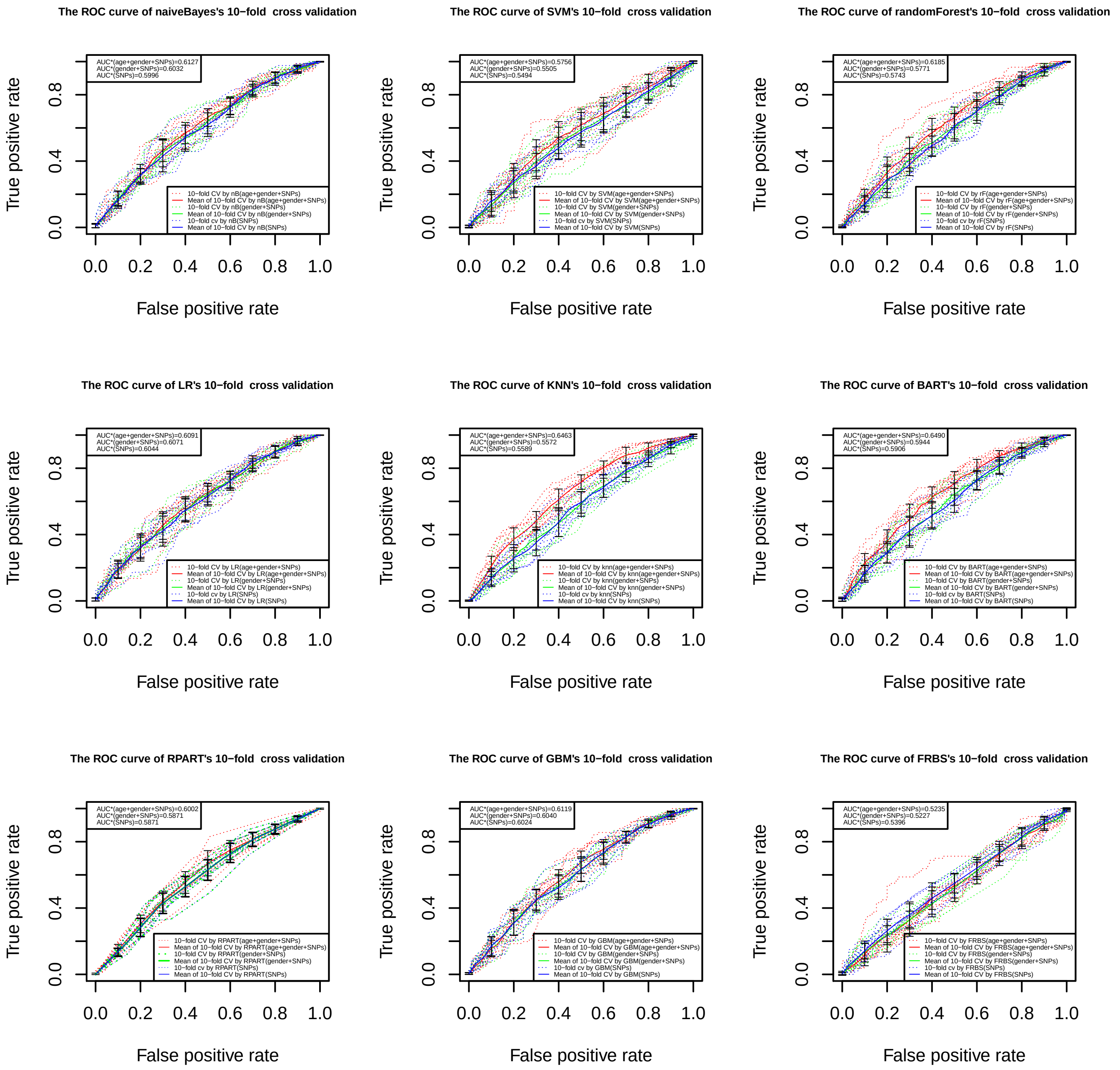

The ROC curve of naiveBayes's 10−fold cross validation
AUC*(age+gender+SNPs)=0.6127
AUC*(gender+SNPs)=0.6032
AUC*(SNPs)=0.5996
10−fold CV by nB(age+gender+SNPs)
Mean of 10−fold CV by nB(age+gender+SNPs)
10−fold CV by nB(gender+SNPs)
Mean of 10−fold CV by nB(gender+SNPs)
10−fold cv by nB(SNPs)
Mean of 10−fold CV by nB(SNPs)
The ROC curve of SVM's 10−fold cross validation
AUC*(age+gender+SNPs)=0.5756
AUC*(gender+SNPs)=0.5505
AUC*(SNPs)=0.5494
10−fold CV by SVM(age+gender+SNPs)
Mean of 10−fold CV by SVM(age+gender+SNPs)
10−fold CV by SVM(gender+SNPs)
Mean of 10−fold CV by SVM(gender+SNPs)
10−fold cv by SVM(SNPs)
Mean of 10−fold CV by SVM(SNPs)
The ROC curve of randomForest's 10−fold cross validation
AUC*(age+gender+SNPs)=0.6185
AUC*(gender+SNPs)=0.5771
AUC*(SNPs)=0.5743
10−fold CV by rF(age+gender+SNPs)
Mean of 10−fold CV by rF(age+gender+SNPs)
10−fold CV by rF(gender+SNPs)
Mean of 10−fold CV by rF(gender+SNPs)
10−fold cv by rF(SNPs)
Mean of 10−fold CV by rF(SNPs)
The ROC curve of LR's 10−fold cross validation
AUC*(age+gender+SNPs)=0.6091
AUC*(gender+SNPs)=0.6071
AUC*(SNPs)=0.6044
10−fold CV by LR(age+gender+SNPs)
Mean of 10−fold CV by LR(age+gender+SNPs)
10−fold CV by LR(gender+SNPs)
Mean of 10−fold CV by LR(gender+SNPs)
10−fold cv by LR(SNPs)
Mean of 10−fold CV by LR(SNPs)
The ROC curve of KNN's 10−fold cross validation
AUC*(age+gender+SNPs)=0.6463
AUC*(gender+SNPs)=0.5572
AUC*(SNPs)=0.5589
10−fold CV by knn(age+gender+SNPs)
Mean of 10−fold CV by knn(age+gender+SNPs)
10−fold CV by knn(gender+SNPs)
Mean of 10−fold CV by knn(gender+SNPs)
10−fold cv by knn(SNPs)
Mean of 10−fold CV by knn(SNPs)
The ROC curve of BART's 10−fold cross validation
AUC*(age+gender+SNPs)=0.6490
AUC*(gender+SNPs)=0.5944
AUC*(SNPs)=0.5906
10−fold CV by BART(age+gender+SNPs)
Mean of 10−fold CV by BART(age+gender+SNPs)
10−fold CV by BART(gender+SNPs)
Mean of 10−fold CV by BART(gender+SNPs)
10−fold cv by BART(SNPs)
Mean of 10−fold CV by BART(SNPs)
The ROC curve of RPART's 10−fold cross validation
AUC*(age+gender+SNPs)=0.6002
AUC*(gender+SNPs)=0.5871
AUC*(SNPs)=0.5871
10−fold CV by RPART(age+gender+SNPs)
Mean of 10−fold CV by RPART(age+gender+SNPs)
10−fold CV by RPART(gender+SNPs)
Mean of 10−fold CV by RPART(gender+SNPs)
10−fold cv by RPART(SNPs)
Mean of 10−fold CV by RPART(SNPs)
The ROC curve of GBM's 10−fold cross validation
AUC*(age+gender+SNPs)=0.6119
AUC*(gender+SNPs)=0.6040
AUC*(SNPs)=0.6024
10−fold CV by GBM(age+gender+SNPs)
Mean of 10−fold CV by GBM(age+gender+SNPs)
10−fold CV by GBM(gender+SNPs)
Mean of 10−fold CV by GBM(gender+SNPs)
10−fold cv by GBM(SNPs)
Mean of 10−fold CV by GBM(SNPs)
The ROC curve of FRBS's 10−fold cross validation
AUC*(age+gender+SNPs)=0.5235
AUC*(gender+SNPs)=0.5227
AUC*(SNPs)=0.5396
10−fold CV by FRBS(age+gender+SNPs)
Mean of 10−fold CV by FRBS(age+gender+SNPs)
10−fold CV by FRBS(gender+SNPs)
Mean of 10−fold CV by FRBS(gender+SNPs)
10−fold cv by FRBS(SNPs)
Mean of 10−fold CV by FRBS(SNPs)
True positive rate
False positive rate
0.0
0.2
0.4
0.6
0.8
1.0
