# Supplementary material for: Significant SNPs have limited prediction ability for thyroid cancer
Source: Cancer Med. 2014 Mar 3;3(3):731–5. doi: 10.1002/cam4.211 (PMC4101765; doi:10.1002/cam4.211)
Supplement: Supplementary file 2 — Table S1. Genomic information for five Acknowledged SNPs from GWAS. Table S2. Model performance with methods based on five SNPs and gender. Table S3. Model performance with methods based on five SNPs, gender, and age. [file cam40003-0731-SD2.docx]

Supplementary Table S1. Genomic information for 5 Acknowledged SNPs from GWAS

| SNP | Coordinate | Alleles | Allele Frequency | Neighbor Genes |
| --- | --- | --- | --- | --- |
| rs965513 | chr9: 100556108 | A,G | 0.212,0.788 | FOXE1, XPA, C9orf156, NCBP1 |
| rs944289 | chr14: 36649245 | C,T | 0.606,0.394 | PTCSC3, LINK00609, BRMS1L |
| rs116909374 | chr14: 36738360 | C,T | 0.979,0.021 | MBIP, PTCSC3, LINK00609, DPPA3 |
| rs966423 | chr2: 218310339 | C,T | 0.716,0.284 | DIRC3 |
| rs2439302 | chr8: 32432368 | C,G | 0.479,0.521 | NRG1 |

The coordination was based on hg19.

Supplementary Table S2. Model performance with methods based on five **SNPs and gender**

|  | AUC | Sensitivity | Specificity | Accuracy | Range of 95% CI of AUC |
| --- | --- | --- | --- | --- | --- |
| K-nearest neighbors | 0.5572 | 0.3958 | 0.6847 | 0.5519 | [0.4225,0.7037] |
| Logistic regression | 0.6071 | 0.4974 | 0.5534 | 0.5281 | [0.4526,0.7492] |
| Naïve Bayes | 0.6032 | 0.4287 | 0.716 | 0.5838 | [0.4648,0.7565] |
| Random Forest | 0.5771 | 0.3326 | 0.7521 | 0.5589 | [0.3793,0.7419] |
| Support vector machine | 0.5505 | 0.2798 | 0.7893 | 0.5557 | [0.4085,0.7305] |
| Bayesian Additive Regression Trees | 0.5944 | 0.4534 | 0.5606 | 0.5124 | [0.4398,0.7279] |
| Boosting | 0.604 | 0.4735 | 0.5553 | 0.5168 | [0.4612,0.7313] |
| Recursive Partitioning | 0.5871 | 0.4085 | 0.7218 | 0.5778 | [0.3926,0.7048] |
| Fuzzy Rule-based system | 0.5227 | 0.4799 | 0.499 | 0.4903 | [0.3603,0.6669] |

AUC, sensitivity, specificity and accuracy were its mean value in 10-fold validations. Range of 95% CI of AUC represents the range of the 95% CI of AUC in 10-fold Cross-validation. SVM represent support vector machines and Kernel Methods

Supplementary Table S3. Model performance with methods based on five **SNPs, gender and age**

|  | AUC | Sensitivity | Specificity | Accuracy | Range of 95% CI of AUC |
| --- | --- | --- | --- | --- | --- |
| K-nearest neighbors | 0.6463 | 0.5062 | 0.6713 | 0.5946 | [0.5117,0.7863] |
| Logistic regression | 0.6091 | 0.5060 | 0.5456 | 0.5276 | [0.4501,0.7441] |
| Naïve Bayes | 0.6127 | 0.4205 | 0.7406 | 0.5941 | [0.4606,0.7415] |
| Random Forest | 0.6185 | 0.4089 | 0.7345 | 0.5838 | [0.4734,0.7976] |
| Support vector machine | 0.5756 | 0.3502 | 0.7744 | 0.5800 | [0.3985,0.7504] |
| Bayesian Additive Regression Trees | 0.6490 | 0.5071 | 0.5786 | 0.5459 | [0.4763,0.7649] |
| Boosting | 0.6119 | 0.4722 | 0.5525 | 0.5146 | [0.4650,0.7340] |
| Recursive Partitioning | 0.6002 | 0.4425 | 0.7014 | 0.5816 | [0.4557,0.7222] |
| Fuzzy Rule-based system | 0.5235 | 0.4960 | 0.5044 | 0.4995 | [0.4152,0.7371] |

AUC, sensitivity, specificity and accuracy were its mean value in 10-fold validations. Range of 95% CI of AUC represents the range of the 95% CI of AUC in 10-fold Cross-validation. SVM represent support vector machines and Kernel Method
